# Association of Albumin-Corrected Serum Calcium Levels with Colorectal Cancer Survival Outcomes

**DOI:** 10.3390/jcm11102928

**Published:** 2022-05-22

**Authors:** Marina Nogueira Silveira, Lara Pozzuto, Maria Carolina Santos Mendes, Lorena Pires da Cunha, Felipe Osório Costa, Lígia Traldi Macedo, Sandra Regina Brambilla, José Barreto Campello Carvalheira

**Affiliations:** Division of Oncology, Department of Anesthesiology, Oncology and Radiology, School of Medical Sciences, State University of Campinas (UNICAMP), Campinas, SP 13083-888, Brazil; marina.nogueira2@gmail.com (M.N.S.); larapozzuto.nutri@gmail.com (L.P.); mariacarol.op@gmail.com (M.C.S.M.); lorenapcunha@yahoo.com (L.P.d.C.); felipeoc@unicamp.br (F.O.C.); ligiamed@gmail.com (L.T.M.); sandraunicamp2@gmail.com (S.R.B.)

**Keywords:** gastrointestinal malignancies, cancer survivorship, calcium carbonate, hypercalcemia, cancer outcomes

## Abstract

In epidemiological studies, higher calcium intake has been associated with decreased colorectal cancer (CRC) incidence. However, whether circulating calcium concentrations are associated with CRC prognosis is largely unknown. In this retrospective cohort analysis, we identified 498 patients diagnosed with stage I–IV CRC between the years of 2000 and 2018 in whom calcium and albumin level measurements within 3 months of diagnosis had been taken. We used the Kaplan–Meier method for survival analysis. We used multivariate Cox proportional hazards regression to identify associations between corrected calcium levels and CRC survival outcomes. Corrected calcium levels in the highest tertile were associated with significantly lower progression-free survival rates (hazard ratio (HR) 1.85; 95% confidence interval (CI) 1.28–2.69; *p* = 0.001) and overall survival (HR 1.86; 95% CI 1.26–2.74, *p* = 0.002) in patients with stage IV or recurrent CRC, and significantly lower disease-free survival rates (HR 1.44; 95% confidence interval (CI) 1.02–2.03; *p* = 0.040) and overall survival rates (HR 1.72; 95% CI 1.18–2.50; *p* = 0.004) in patients with stage I–III disease. In conclusion, higher corrected calcium levels after the diagnosis of CRC were significantly associated with decreased survival rates. Prospective trials are necessary to confirm this association.

## 1. Introduction

Every year, it is expected that approximately 1.88 million people will be diagnosed with colorectal cancer (CRC) worldwide, while 915,880 deaths are attributed to the disease [1]. Although current changes in risk factors such as decreased smoking and red meat consumption may have contributed to the decline in overall incidence of CRC in some countries, it is still the third most common type of cancer [2]. For this reason, research that considers possible predictive as well as prognostic factors is needed.

Higher calcium intake has been associated with a decreased risk of CRC [3,4,5]. Results from a meta-analysis of 21 publications showed that for each 300 mg of calcium consumed, there was a reduction of 8–9% in the risk of acquiring CRC [6]. Consistently, a pooled analysis of 534,536 individuals also revealed a reduction in CRC with higher calcium intake [7]. In contrast, a recently published, large prospective trial found that calcium and calcitriol supplementation was associated with an increased risk of the development of serrated polyps, a CRC precursor lesion [8]. Although calcium signaling is a key player in the fundamental stages of cancer development, the complexities of calcium intersections with oncogenic pathways are context dependent (i.e., the alignment of calcium channels in cancer cells, extracellular calcium concentrations, and calcium interactions with the microenvironment are factors that determine calcium influence in cancer cell fate) [9]. Moreover, increased levels of extracellular calcium are insufficient to modulate cancer cell proliferation. Rather, cytosolic calcium levels, which are mainly determined by the activity of calcium channels, pumps, and exchangers, are key to the control of intracellular calcium levels in a context-dependent manner [9]. Thus, it is simple to delineate a plausible biological framework of how the modulation of extracellular calcium levels by calcium intake influences carcinogenesis.

Much less is known about the effects of serum calcium on the risk of developing CRC. In striking opposition to the association between calcium intake and a reduced risk of CRC, a retrospective analysis of a Swedish databank (Apolipoprotein Mortality Risk (AMORIS)) showed a modest increase in the risk of developing CRC in the highest quartiles of albumin-corrected serum calcium [10]. Calcium homeostasis is regulated not only by calcium intake but also by bidirectional fluxes of this ion at the level of the kidneys and bones [11]. Importantly, calcium homeostasis is disrupted in 20–30% of patients during the course of cancer development [12], making it biologically plausible that the association between higher calcium levels and CRC observed in the AMORIS study may be related to calcium homeostasis disturbances mediated by the initial stages of tumor development.

In normal physiology, extracellular calcium levels are mainly regulated by parathyroid hormone (PTH), calcitonin, and calcitriol [11]. On the other hand, in cancer pathology, the vast majority of hypercalcemia of malignancy is associated with increased PTH-related peptide (PTHrP) levels [12]. Interestingly, PTHrP has a limited role during cartilage embryogenesis through the activation of the hedgehog signaling pathway in cartilage cells [13,14]. In cancer cells, this dormant pathway is reactivated, increasing circulating levels of PTHrP [15], which leads to the elevation of the extracellular calcium concentration in a tumor-burden- and aggressiveness-dependent manner [16,17]. Thus, bearing in mind the hypothesis that the corrected calcium level is a biomarker of cancer progression, we sought to examine the impact of corrected calcium levels in the outcomes of patients with CRC, using both calcium and albumin levels routinely measured during clinic visits reported in medical records.

## 2. Materials and Methods

### 2.1. Study Population

**Figure 1:**
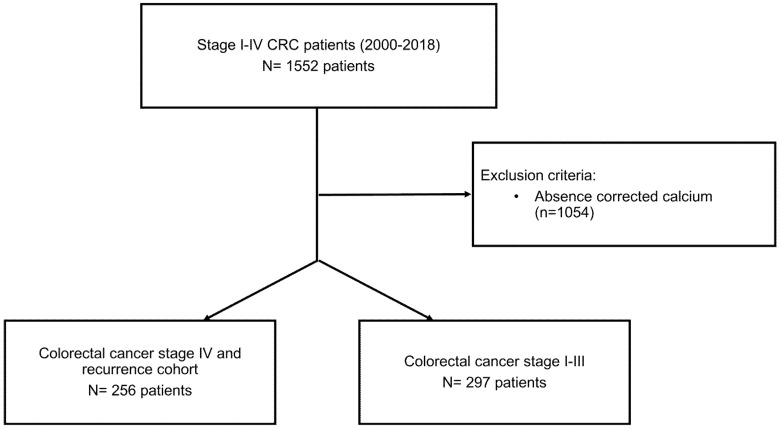
Study flowchart.

This study was a single-center, retrospective, and analytical study conducted at the State University of Campinas Hospital (HC-UNICAMP) in Campinas, Brazil. The study population was composed of patients diagnosed with stage I–IV CRC between the years of 2000 and 2018, admitted to the HC-UNICAMP. Patients that met the following inclusion criteria were selected: histologically confirmed CRC between 2000 and 2018; CRC stage I–IV according to the 8th edition of the American Joint Committee on Cancer (AJCC) cancer staging manual [18]; the availability of calcium and albumin measurements within 3 months of the diagnosis for stage IV or recurrent CRC; the availability of calcium and albumin measurements before surgery in patients with stage I–III CRC; and complete medical record information regarding age, date of diagnosis, topography, histological type, and tumor staging. Patients with concomitant malignancies, CRC that was not adenocarcinoma, in situ CRC, or unreported data regarding treatment were excluded (Figure 1). 

The study was approved by the local Institutional Review Board (CAAE number: 15505419.1.0000.5404) with a consent form waiver. The principles recommended by the Declaration of Helsinki were adhered to.

### 2.2. Body Composition

Two consecutive computed tomography (CT) images of the third lumbar vertebra were evaluated; the images were obtained from routine examinations of the patients. Baseline imaging was performed within 3 months of diagnosis for patients with stage I–III CRC and 3 months before diagnosis or chemotherapy initiation for patients with stage IV or recurrent CRC. Skeletal muscle (SM) values of the psoas, abdominal, rectus abdominis, and paravertebral muscles were measured [19,20]. The visceral adipose tissue (VAT), intramuscular adipose tissue (IMAT), and subcutaneous adipose tissue (SAT) were also measured; from these values, we determined the SM index (SMI), the SAT index (SFI), and the VAT index (VFI), measured in units of square centimeters (cm²) and normalized by height in square meters (m²). The software used was SliceOmatic V. 5.0. (Tomovision, Canada); standard Hounsfield units (HUs) established for tissues were −150 to −50 for VAT, −190 to −30 for IMAT and SAT, and −29 to 150 for SM. The images were analyzed by two evaluators (M.N.S. and L.P.) blinded to the outcomes, and the coefficients of variation for the cross-sectional areas analyzed were 1.07%, 1.05%, 1.61%, and 3.57% for SM, SAT, VAT, and IMAT, respectively, and 1.60% for SM density.

### 2.3. Data Collection

Data were collected from medical records, specifically from the time of CRC diagnosis until the date of death or last follow-up. Research Electronic Data Capture (REDCap) software was used for the construction of case report forms (CRFs) and database management [21].

#### 2.3.1. Clinical Variables

The variables collected comprised sociodemographic characteristics (age, sex, ethnicity, smoking status, and alcohol use status) and anthropometric characteristics (weight, weight loss (WL), height, and body mass index (BMI) at diagnosis). Additionally, disease-related covariates were obtained regarding the date of CRC diagnosis, the Eastern Cooperative Oncology Group Performance Status Scale (ECOG) status, chemotherapy regimens, the primary tumor location, the Charlson Comorbidity Index [22], carcinoembryonic antigen (CEA), the number of metastases, emergency surgery, and the clinical and pathological stage according to the AJCC cancer staging manual (tumor, node, and metastasis (TNM)) [23].

#### 2.3.2. Biochemical Exam Data

Serum albumin and calcium levels were measured using calorimetric assays according to the HC-UNICAMP clinical pathology protocol. Calcium (mg/dL; reference range: 8.8–10.2 mg/dL for adults aged 21–50 years and 8.4–9.7 mg/dL for adults > 50 years old), albumin (mg/dL; reference range: 3.4–4.8 g/dL), baseline CEA levels (ng/mL; cut-off value: 5 ng/mL), and complete blood count levels were collected within 3 months of diagnosis for patients with stage IV or recurrent CRC, and before surgery for patients with stage I–III CRC. 

#### 2.3.3. Corrected Calcium Measurement

Given that ionized calcium is not measured routinely in clinics, we used corrected calcium to estimate the free calcium concentration, which was calculated using the following formula: corrected calcium = serum calcium + [(4.0 − serum albumin) × 0.8] [24]. The corrected calcium levels were categorized into tertiles.

#### 2.3.4. Systemic Inflammatory Indexes

The neutrophil-to-lymphocyte ratio (NLR) was calculated by dividing the absolute count of neutrophils by the absolute count of lymphocytes [25]. The platelet-to-lymphocyte ratio (PLR) was calculated by dividing the absolute count of platelets by the absolute count of lymphocytes [26]. The lymphocyte-to-tomonocyte ratio (LMR) was calculated by dividing the absolute count of lymphocytes by the absolute count of monocytes. The NLR and PLR were analyzed as continuous variables.

#### 2.3.5. Endpoints

The co-primary endpoints were progression-free survival and overall survival, which were calculated using the time between disease diagnosis or recurrence and the first event (disease progression or death) and death from any cause, respectively. Data regarding mortality were obtained from medical records. To evaluate the outcomes, the last date of follow-up recorded in the medical record or the date of death of the patient was considered.

### 2.4. Statistical Analysis

After summarizing the baseline characteristics based on the corrected calcium levels using descriptive statistics, the characteristics were compared by using chi-square and Kruskal–Wallis tests. Multivariate-adjusted Cox proportional hazards regression models were used to investigate associations between corrected calcium and progression-free and overall survival. Time was calculated in months from the diagnosis to the time of the event or the last follow-up visit (through August 2018).

To minimize the effects of potential confounders in our regression model, we included variables related to CRC-specific mortality outcomes established in previous studies. We also included variables that were associated (*p* < 0.10 in the unadjusted Cox analysis) with CRC mortality. We used the Kaplan–Meier method for survival analysis.

Analyses stratified by stage, cancer site, age, and gender were performed. Two-sided *p* values < 0.05 were considered to be statistically significant. The STATA 12 software was used for statistical analysis.

## 3. Results

### 3.1. Patient Disposition and Baseline Characteristics

A total of 256 patients with stage IV or recurrent CRC were included in our study; 207 died of any cause, with a median follow-up of 15.7 months (interquartile range (IQR) 5.8–32.6 months) at the time of the analysis.

Baseline characteristics according to corrected calcium levels are shown in Table 1. Generally, subjects with high levels of corrected calcium (≥9.46 mg/dL) were younger, had more metastases, the highest CEA levels, were less often submitted to prior neoadjuvant or adjuvant treatment, and were less often submitted to a backbone chemotherapy regimen with oxaliplatin. The other characteristics evaluated were similar among the calcium levels.

We also evaluated 243 patients with CRC stage I–III for calcium levels < 9.44 mg/dL and ≥9.44 mg/dL; only sex correlated with higher calcium levels (*p* = 0.002) (Table S1).

### 3.2. Body Composition and Inflammatory Indexes

The serum calcium levels of patients with stage IV or recurrent CRC showed no correlation with body composition variables; however, when evaluating inflammatory markers, there were higher levels of the NLR and the PLR (Table S2).

In non-metastatic patients, serum calcium levels ≥ 9.44 mg/dL correlated with lower IMAT (*p* = 0.016), lower NLR (*p* = 0.005), and higher LMR (*p* = 0.025) (Table S3).

### 3.3. Survival Analysis

**Figure 2:**
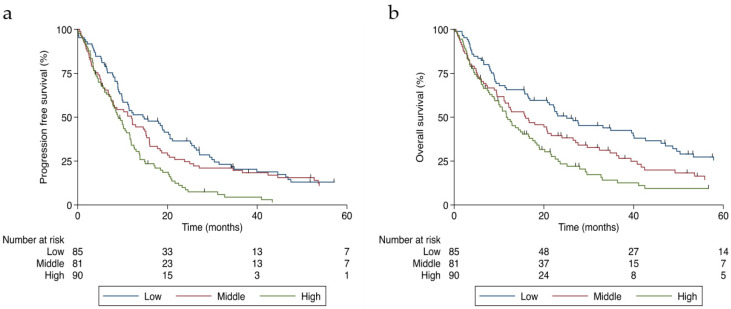
Survival curves of patients with stage IV or recurrent colorectal cancer divided by corrected calcium tertiles (in mg/dL): (**a**) progression-free survival and (**b**) overall survival.

As shown in Table 2, unadjusted Cox regression revealed that higher levels of corrected calcium were associated with reduced median progression-free survival (*p* < 0.001) and overall survival (*p* < 0.001) rates in patients with stage IV or recurrent CRC. The significant association persisted after adjusting the model for age, BMI, ECOG, baseline CEA levels, the number of metastases, chemotherapy use, and WL. High levels of calcium were associated with decreased median progression-free survival (hazard ratio (HR) 1.85; 95% confidence interval (CI) 1.27–2.69, *p* = 0.001) (Figure 2a) and overall survival rates (HR 1.86; 95% CI 1.26–2.74, *p* = 0.002) (Figure 2b).

**Figure 3:**
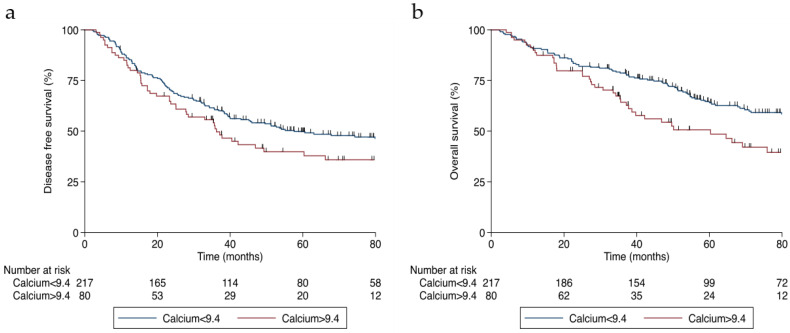
Survival curves of patients with stage I–III colorectal cancer divided by corrected calcium levels (in mg/dL): (**a**) disease-free survival and (**b**) overall survival.

Likewise, higher levels of calcium were associated with decreased progression-free survival (HR 1.44; 95% CI 1.02–2.03, *p* = 0.040) (Figure 3a) and overall survival rates (HR 1.72; 95% CI 1.18–2.50, *p* = 0.004) (Figure 3b) in patients with stage I–III CRC, even after adjusting the model for age, BMI, WL, smoking, the Charlson Comorbidity Index, cancer stage, and emergency surgery.

## 4. Discussion

In this retrospective cohort, patients with metastatic disease presented with significantly decreased progression-free and overall survival rates in a corrected-calcium-dependent manner. Importantly, this association appeared to be independent of age, BMI, ECOG, CEA levels, the number of metastases, chemotherapy regimen, and WL. Moreover, patients with non-metastatic disease had increased risk of progression and mortality even after adjusting the model for age, BMI, WL, smoking, the Charlson Comorbidity Index, and emergency surgery. However, our analysis with body composition was not associated with corrected calcium levels.

Previous studies have evaluated the effects of hypercalcemia of malignancy (calcium greater than upper limit of normal (ULN)) on disease outcomes but not the influence of calcium as a biomarker of CRC progression. These reports consistently associate hypercalcemia with a poor prognosis [27,28]. Thus, a key question is that beyond corrected calcium directly influencing cancer outcomes, is it also a biomarker of cancer progression?

Interestingly, a few reports have investigated the role of corrected calcium as a marker of cancer progression without categorizing it in the ULN. For example, in the setting of metastatic kidney cancer, the set level of corrected calcium commonly used in prognostic models of survival (a tool used routinely in clinics) is lower than the ULN [29,30]. Interestingly, extracellular calcium levels per se have an established CRC chemoprotective effect [31,32,33]. Extracellular calcium levels may be considered a marker for increased PTH and vitamin D levels. Like extracellular calcium levels, serum vitamin D level is associated with reduced cancer mortality [34]; thus, a possible biological explanation for the association of corrected calcium levels with cancer progression is that corrected calcium may reflect the spectrum of PTHrP secreted by the tumor. PTHrP is expressed in >90% of CRC cases, and the grade of its expression was previously correlated with poor differentiation and aggressiveness [17]. We also found an association between higher levels of corrected calcium and a greater number of metastases and higher levels of CEA, which are important prognostic biomarkers for metastatic CRC. Consistent with this finding, PTHrP was recently linked to the increased proliferation of colon cancer cells [35]. Moreover, elevated PTHrP levels have been associated with cancer cachexia [16]. However, in our analysis, body composition, excluding IMAT in nonmetastatic patients, was not associated with corrected calcium, corroborating the findings of a recent study where the serum PTHrP level was not correlated with WL, uncoupling protein (UCP)-1, and other white adipose tissue browning markers [36]. Thus, additional prospective studies are needed to elucidate the role of PTHrP in determining body composition in humans.

Interestingly, in a cohort of patients with gastroesophageal cancer, the levels of PTHrP were associated with poor prognosis independently of overt hypercalcemia. Hence, one could assume that PTHrP interferes with calcium levels within the normal range [37]. In accordance, the antibody neutralization of PTHrP in mice bearing tumors improved survival [38]. In another animal model, PTHrP showed great influence on tumorigenesis, progression, and metastasis formation in breast cancer xenografts [39]. Moreover, Carriere et al. [40] recently suggested the involvement of PTHrP, secreted protein acidic and rich in cysteine (SPARC), and epithelial–mesenchymal transition (EMT) in CRC, favoring a more aggressive phenotype of the disease. Consistent with the idea of an unmediated effect of calcium levels on survival outcomes, our assessment of inflammatory indexes revealed the opposite results. While patients with stage IV or recurrent CRC with higher calcium levels had greater inflammation, in patients with local and locoregional disease, higher calcium levels were associated with lower inflammatory rates. This suggests that calcium levels do not modulate the inflammatory milieu of the host; rather, the higher calcium levels observed in metastatic patients may be a consequence of the greater tumor burden.

The strength of our study is that it is the first to separately report this association in patients with both locoregional and advanced CRC. Nonetheless, our study has limitations. First, the retrospective nature of this study impeded any further analysis of the mechanisms involved in the association of corrected calcium with prognosis, such as the measurement of PTHrP. Second, the generalizability of our study is limited. We conducted our study in a single institution, and patients attended HC-UNICAMP; these patients represent a population in São Paulo that does not have private insurance and thus does not represent the higher socioeconomic spectra. Third, although the calcium and albumin measurements were obtained in a manner that was dependent on the assistant physician’s choice, the notable number of missing data points in our cohort (of the 1552 patients in this cohort, only 498 had calcium and albumin measurements at the given time) may have potentiated unrecognized changes in clinical practice during the study timeframe. Finally, our findings need to be tested in future studies that examine other populations, which must include information regarding PTHrP, calcium, PTH, and vitamin D levels.

In summary, we demonstrated that higher corrected calcium levels might be associated with worse CRC survival outcomes. Although reverse causality may have contributed to our findings, the use of corrected calcium levels as a biomarker of CRC prognosis holds promise for better understanding the mechanisms of CRC aggressiveness and deserves further evaluation in prospective trials to be implemented as a prognostic predictor in clinical practice.

## Figures and Tables

**Table 1:** Selected demographic and clinical characteristics and laboratory findings according to calcium tertiles of patients with metastatic colorectal cancer.

		Corrected Calcium, mg/dL			
Characteristic	All Patients, *n* = 256	Low Tertile, *n* = 85 7.18–8.98	Middle Tertile, *n* = 81 9.00–9.44	High Tertile, *n* = 90 9.46–14.24	*p*
Age (years), number (%)					
<55	93 (36.3)	26 (30.6)	24 (29.6)	43 (47.8)	0.039 ^a^
55–70	112 (43.8)	37 (43.5)	39 (48.2)	36 (40.0)	
>70	51 (19.9)	22 (25.9)	18 (22.2)	11 (12.2)	
Sex, number (%)					
Male	150 (58.6)	56 (65.9)	45 (55.6)	49 (54.4)	0.246 ^a^
Female	106 (41.4)	29 (34.1)	36 (44.4)	41 (45.6)	
BMI (kg/m^2^), number (%)					
<18.5	24 (9.4)	7 (8.3)	5 (6.2)	12 (13.3)	0.682 ^a^
18.5–24.9	135 (52.7)	45 (52.9)	41 (50.6)	49 (54.4)	
25–29.9	63 (24.6)	22 (25.9)	22 (27.2)	19 (21.1)	
≥30	34 (13.3)	11 (12.9)	13 (16.0)	10 (11.1)	
Weight loss, number (%)					
<5%	69 (27)	30 (35.3)	21 (25.9)	18 (20.0)	0.233 ^a^
5–10%	39 (15.2)	10 (11.8)	13 (16.1)	16 (17.8)	
>10%	148 (57.8)	45 (52.9)	47 (58.0)	56 (62.2)	
Active smoker, number (%)	116 (45.9)	40 (47.6)	38 (47.5)	38 (42.7)	0.760 ^a^
Active alcohol user, number (%)	81 (31.9)	31 (36.5)	28 (34.6)	22 (25.0)	0.222 ^a^
Topography, number (%)					
Left	208 (81.2)	70 (82.3)	68 (84)	70 (77.8)	0.558 ^a^
Right	48 (18.8)	15 (17.7)	13 (16.0)	20 (22.2)	
ECOG, number (%)					
0	209 (90.5)	71 (94.7)	67 (93.1)	71 (84.5)	0.070 ^b^
I	22 (9.5)	4 (5.3)	5 (6.9)	13 (15.5)	
II					
Stage, number (%)					
I–II	33 (12.9)	15 (17.7)	12 (14.8)	6 (6.7)	0.026 ^b^
III	21 (8.2)	8 (9.4)	10 (12.3)	3 (3.3)	
IV	202 (78.9)	62 (72.9)	59 (72.8)	81 (90.0)	
Metastasis, number (%)					
1	143 (55.9)	54 (63.5)	44 (54.3)	45 (50.0)	0.017 ^b^
2 or more	104 (40.6)	26 (30.6)	33 (40.7)	45 (50.0)	
Local recurrence	9 (3.5)	5 (5.9)	4 (4.9)	0 (0.0)	
CEA (ng/mL), median (IQR)	30.9 (6.37–157.3)	20.1 (4.6–74.4)	26.9 (4.4–122.9)	87.6 (11.2–406.0)	<0.001 ^c^
Prior neoadjuvant or adjuvant treatment, number (%)	81 (31.6)	34 (40.0)	31 (38.3)	16 (17.8)	0.002 ^a^
Bevacizumab containing regimen, number (%)	53 (27.0)	14 (21.9)	20 (33.9)	19 (26.0)	0.315 ^a^
Backbone chemotherapy regimen, number (%)					
Oxaliplatin	62 (32.8)	25 (40.3)	21 (36.2)	16 (23.2)	0.016 ^a^
Irinotecan	105 (55.6)	33 (53.2)	34 (58.6)	38 (55.1)	
5-Fluorouracil	22 (11.6)	4 (6.5)	3 (5.2)	15 (21.7)	

Abbreviations: BMI: body mass index; CEA: carcinoembryonic antigen; ECOG: Eastern Cooperative Oncology Group Performance Scale; IQR: interquartile range. ^a^ Chi-square test, ^b^ Fisher’s exact test, ^c^ Kruskal–Wallis test.

**Table 2:** Corrected calcium and survival for patients with metastatic colorectal cancer.

	Corrected Calcium, mg/dL [HR (95% CI)]			
	Low Calcium	Middle Calcium	High Calcium	*p*
	(7.18–8.98)	(9.00–9.44)	(9.46–14.24)	
Progression-free survival				
Number of events/at risk	68/85	74/81	84/90	
Unadjusted	Referent	1.27 (0.91–1.76)	1.94 (1.41–2.69)	<0.001
Adjusted ^a^	Referent	1.17 (0.80–1.71)	1.85 (1.27–2.69)	0.001
Overall survival				
Number of events/at risk	63/85	67/81	77/90	
Unadjusted	Referent	1.44 (1.02–2.04)	1.98 (1.41–2.79)	<0.001
Adjusted ^a^	Referent	1.16 (0.78–1.74)	1.86 (1.26–2.74)	0.002

Abbreviations: CI, confidence interval; HR, hazard ratio. ^a^ Cox model adjusted for age, body mass index, Eastern Cooperative Oncology Group Performance Scale, number of metastases, chemotherapy use, and weight loss.

## Data Availability

Not applicable.

## Supplementary Materials

The following supporting information can be downloaded at: https://www.mdpi.com/article/10.3390/jcm11102928/s1, Table S1: Selected demographic and disease characteristics according to calcium levels of stage I–III colorectal cancer patients; Table S2: Body composition and inflammatory indexes according to calcium tertiles of metastatic colorectal cancer patients; Table S3: Body composition and inflammatory indexes according to calcium tertiles of stage I–III colorectal cancer patients
