# Supplementary material for: Association of Albumin-Corrected Serum Calcium Levels with Colorectal Cancer Survival Outcomes
Source: J Clin Med. 2022 May 22;11(10):2928. doi: 10.3390/jcm11102928 (PMC9144533; doi:10.3390/jcm11102928)
Supplement: Supplementary file 1 [file jcm-11-02928-s001.zip › 20220228_Supplementary_Table_S1.zip!/20220228_Supplementary_Table_S1.pdf]

Supplemental Table S1. Selected demographic and disease characteristics according to calcium levels of stage I-III colorectal cancer patients.

Characteristic	All-patients, n=297	Calcium <9.44 mg/dl n=217	Calcium >9.44 mg/dl n=80	P value
**Age, № (%)**				
<55	80 (26.9)	63 (29.0)	17 (21.3)	0.377[a]
55-70	137 (46.1)	96 (44.2)	41 (51.3)	
>70	80 (26.9)	58 (26.7)	22 (27.5)	
**Sex, № (%)**				
Female	155 (52.2)	125 (57.6)	30 (37.5)	0.002[a]
Male	142 (47.8)	92 (42.4)	50 (62.5)	
**BMI, median (IQR)**				
<18.5	20 (6.7)	16 (7.4)	4 (5.0)	0.763[a]
18.5-24.9	125 (42.1)	88 (40.6)	37 (46.3)	
25-29.9	96 (32.3)	70 (32.3)	26 (32.5)	
≥30	56 (18.9)	43 (19.8)	13 (16.3)	
**Weight loss, № (%)**				
<5%	112 (38.4)	84 (39.3)	28 (35.9)	0.233[a]
5-10%	79 (27.1)	58 (27.1)	21 (26.9)	
>10%	101 (34.6)	72 (33.6)	29 (37.2)	
**Alcohol consumption, № (%)**	129 (43.7)	90 (41.9)	39 (48.8)	0.289[a]
**Smoking, № (%)**	91 (30.9)	67 (31)	24 (30.4)	0.216[a]
**Charlson comorbidity index, № (%)**				
2-3	105 (35.4)	83 (38.3)	22 (27.5)	0.086[a]
≥ 4	192 (64.6)	134 (61.7)	58 (72.5)	
**Stage, № (%)**				
I-II	166 (55.9)	124 (57.1)	42 (52.5)	0.706[b]
III	115 (38.7)	82 (37.8)	33 (41.3)	
TXNXM0	16 (5.4)	11 (5.1)	5 (6.3)	
**CEA, Median (IQR)**	4.5 (2.2–13.0)	4 (2.2–13.6)	5.0 (2.3–10.6)	0.719[c]
**Emergency surgery, № (%)**	24 (8.1)	20 (9.2)	4 (5.1)	0.338[b]
**Neoadjuvant treatment, № (%)**	213 (71.7)	151 (69.6)	62 (77.5)	0.179[a]
**Adjuvant treatment, № (%)**	176 (59.5)	133 (61.3)	43 (54.4)	0.288[a]

Abbreviations: BMI: Body mass index; CEA: carcinoembryonic antigen; IQR: interquartile range
[a]Chi square test, [b]Fisher's exact test, [c]Mann Whitney's test.
