# Supplementary material for: Association of Albumin-Corrected Serum Calcium Levels with Colorectal Cancer Survival Outcomes
Source: J Clin Med. 2022 May 22;11(10):2928. doi: 10.3390/jcm11102928 (PMC9144533; doi:10.3390/jcm11102928)
Supplement: Supplementary file 1 [file jcm-11-02928-s001.zip › 20220228_Supplementary_Table_S2.zip!/20220228_Supplementary_Table_S2.pdf]

Supplemental Table S2. Body composition and inflammatory indexes according to calcium tertiles of metastatic colorectal cancer patients.

Characteristic	All-patients, n=79	Low tertile, n=28 7.18–8.98	Middle tertile, n=29 9.00–9.44	High tertile, n=22 9.46–14.24	P value
**Skeletal Muscle**					
Area (cm^2), mean (SD)	130.1 (30.8)	129.8 (30.4)	134.5 (32.4)	124.6 (29.8)	0.908[a]
SMI (cm^2/m^2)), mean (SD)	47.8 (9.2)	47.9 (10.4)	49.5 (8.8)	45.5 (7.8)	0.370[a]
Attenuation (HU)), mean (SD)	32.6 (9.8)	33.7 (9.1)	30.4 (9.5)	34.0 (11.1)	0.579[a]
IMAT, area (cm^2), median (IQR)	9.0 (5.6–13.4)	8.5 (5.5–11.1)	10.0 (7.2–17.1)	7.6 (5.5–11.0)	0.152[b]
**Visceral adipose tissue**					
VAT, area (cm^2), median (IQR)	81.1 (45.0–138.8)	76.1 (37.9–136.2)	117.4 (53.4–158.7)	80.8 (41.9–118.4)	0.320[b]
VATI (cm^2/m^2)), median (IQR)	29.8 (17.9–48.4)	28.3 (14.6–47.1)	38.8 (19.2–58.5)	29.8 (14.3–40.5)	0.320[b]
VAT attenuation (HU), median (IQR)	-90.8 (-97.2 to -83.3)	-90.2 (-97.1 to -82.0)	-92.4 (-99.5 to -84.8)	-91.0 (-96.7 to -83.3)	0.524[b]
**Subcutaneous adipose tissue**					
SAT, area (cm^2), median (IQR)	129.9 (81.7–214.2)	126.3 (82.6–180.9)	134.6 (86.6–239.1)	112.2 (77.6–241.0)	0.638[b]
SATI (cm^2/m^2), median (IQR)	48.4 (29.7–73.7)	48.3 (28.7–64.7)	52.1 (32.6–86.8)	43.8 (29.7–88.1)	0.615[b]
SAT attenuation (HU), median (IQR)	-98.6 (-106.8 to -90.7)	-97.2 (-106.4 to -88.7)	-101.4 (-106.8 to -92.7)	-98.2 (-104.7 to -90.7)	0.743[b]
**Characteristic**	**All-patients, n=253**	**Low tertile, n=84 7.18–8.98**	**Middle tertile, n=80 9.00–9.44**	**High tertile, n=89 9.46–14.24**	**P value**
**Inflammatory indexes**					
NLR, median (IQR)	3.3 (2.2–5.1)	2.7 (1.9–4.1)	3.3 (2.2–4.7)	3.9 (2.5–6.5)	**0.002**[b]
PLR, median (IQR)	173.4 (128.1–276.4)	165.1 (111.3–231.8)	170.3 (128.7–243.3)	224.0 (137.6–340.1)	**0.004**[b]
LMR, median (IQR)	2.6 (1.8–3.6)	2.8 (1.9–4.0)	2.5 (1.8–3.6)	2.2 (1.7–3.1)	0.051[b]

Abbreviations: IMAT: Intramuscular adipose tissue; IQR: interquartile range; LMR: lymphocyte-to-monocyte ratio; NLR: neutrophil-to-lymphocyte ratio; PLR: platelet-to-lymphocyte SAT: subcutaneous adipose tissue; SATI: Subcutaneous Fat Index; SD: Standard deviation; SMI: Skeletal Muscle Index; VAT: Visceral adipose tissue; VATI: Visceral Fat Index. [a]ANOVA oneway test, [b]Kruskal Wallis's test.
