# Supplementary material for: Association of Albumin-Corrected Serum Calcium Levels with Colorectal Cancer Survival Outcomes
Source: J Clin Med. 2022 May 22;11(10):2928. doi: 10.3390/jcm11102928 (PMC9144533; doi:10.3390/jcm11102928)
Supplement: Supplementary file 1 [file jcm-11-02928-s001.zip › 20220228_Supplementary_Table_S3.zip!/20220228_Supplementary_Table_S3.pdf]

Supplemental Table S3. Body composition and inflammatory indexes according to calcium tertiles of stage I-III colorectal cancer patients.

Characteristic	All-patients, n=125	Calcium <9.44 mg/dl n=94	Calcium >9.44 mg/dl n=31	P value
**Skeletal Muscle**				
Area (cm^2), mean (SD)	129.5 (35.6)	131.6 (36.4)	123.1 (32.4)	0.252[a]
SMI (cm^2/m^2)), mean (SD)	49.3 (10.9)	49.8 (11.1)	47.7 (10.5)	0.363[a]
Attenuation (HU)), mean (SD)	32.4 (9.5)	32.0 (9.5)	33.7 (9.6)	0.382[a]
IMAT, area (cm^2), median (IQR)	8.1 (4.7–10.0)	10.4 (6.9–15.9)	9.2 (6.2–15.0)	**0.016**[b]
**Visceral adipose tissue**				
VAT, area (cm^2), median (IQR)	125.8 (62.2–191.3)	130.9 (65.5–212.0)	119.6 (49.4–168.3)	0.121[b]
VATI (cm^2/m^2)), median (IQR)	50.9 (25.7–75.0)	53.6 (26.4–80.3)	42.7 (21–63.1)	0.179[b]
VAT attenuation (HU), median (IQR)	-96.1 (-101.7 to -84.8)	-96.5 (-102.7 to -84.8)	-94.0 (-98.8 to -85.2)	0.339[b]
**Subcutaneous adipose tissue**				
SAT, area (cm^2), median (IQR)	129.7 (87.5–191.1)	131.2 (85.1–198.0)	127.0 (87.5–182.0)	0.810[b]
SATI (cm^2/m^2), median (IQR)	46.0 (33.8–77.9)	45.6 (33.0–79.7)	48.4 (34.7–75.7)	0.914[b]
SAT attenuation (HU), median (IQR)	-98.3 (-105.4 to -89.9)	-98.6 (-105.8 to -92.1)	-96.7 (-102.1 to -87.6)	0.278[b]
**Characteristic**	**All-patients, n=295**	**Calcium <9.44 mg/dl n=216**	**Calcium >9.44 mg/dl n=79**	**P value**
**Inflammatory indexes**				
NLR, median (IQR)	2.4 (1.7–3.8)	2.6 (1.8–4.0)	2.1 (1.5–2.8)	**0.005**[b]
PLR, median (IQR)	137.7 (106.9–202.0)	140.0 (106.9–206.1)	133.9 (106.8–197.2)	0.501[b]
LMR, median (IQR)	3.2 (2.2–4.4)	3.0 (2–4.3)	3.5 (2.5–4.4)	**0.025**[b]

Abbreviations: IMAT: Intramuscular adipose tissue; IQR: interquartile range; LMR: lymphocyte-to-monocyte ratio; NLR: neutrophil-to-lymphocyte ratio; PLR: platelet-to-lymphocyte SAT: subcutaneous adipose tissue; SATI: Subcutaneous Fat Index; SD: Standard deviation; SMI: Skeletal Muscle Index; VAT: Visceral adipose tissue; VATI: Visceral Fat Index. [a]Student's T test, [b]Mann Whitney's test.
